# Shared decision-making in physiotherapy: a cross-sectional study of patient involvement factors and issues in Japan

**DOI:** 10.1186/s12911-023-02208-1

**Published:** 2023-07-24

**Authors:** Tatsuya Ogawa, Shuhei Fujimoto, Kyohei Omon, Tomoya Ishigaki, Shu Morioka

**Affiliations:** 1 Department of Neurorehabilitation, Graduate School of Health Sciences, Kio University, 4-2-2 Umaminaka, Koryo-Cho, Kitakatsuragi-Gun, Nara, 635-0832 Japan; 2 Department of Rehabilitation, Nishiyamato Rehabilitation Hospital, 3-2-2 Sasayuridai, Kanmaki-Cho, Kitakatsuragi-Gun, Nara, 639-0218 Japan; 3 Kyoto University Graduate School of Public Health, Yoshida-Honmachi, Sakyo-Ku, Kyoto, 606-8501 Japan; 4 Shizuoka Graduate University of Public Health, 4-27-2 Kitaando, Aoi-Ku, Shizuoka-Shi, Shizuoka, 420-0881 Japan; 5 Rehabilitation Center, Kishiwada Rehabilitation Hospital, 2-8-10 Kanmatsu-Cho, Kishiwada-Shi, Osaka, 596-0827 Japan; 6 Department of Cognitive Behavioral Science, Kyoto University Graduate School of Human and Environmental Studies, Yoshida-Nihonmatsucho, Sakyo-Ku, Kyoto City, 606-8501 Japan; 7 Department of Physical Therapy, Faculty of Rehabilitation, Nagoya Gakuin University, 3-1-17 Taiho, Atsuta-Ku, Nagoya-Shi, Aichi, 456-0062 Japan; 8 Department of Rehabilitation, Kawaguchi Neurosurgery Rehabilitation Clinic, 9-25-202 Kourien-Cho, Hirakata-Shi, Osaka, 573-0086 Japan

**Keywords:** Shared decision making, Physiotherapy, Rehabilitation, Preference, Patient involvement, Patient participation

## Abstract

**Background:**

Evidence-based medicine education has not focused on how clinicians involve patients in decision-making. Although shared decision-making (SDM) has been investigated to address this issue, there are insufficient data on SDM in physiotherapy. This study aimed to clarify the issues concerning patient involvement in Japan, and to examine whether SDM is related to perceptions of patient involvement in decision-making.

**Methods:**

The study participants were recruited from among acute and sub-acute inpatients and community residents receiving physiotherapy outpatient care, day care, and/or home rehabilitation. The Control Preference Scale (CPS) was used to measure the patients' involvement in decision-making. The nine-item Shared Decision-Making Questionnaire (SDM-Q-9) was used to measure SDM. In analysis I, we calculated the weighted kappa coefficient to examine the congruence in the CPS between the patients' actual and preferred roles. In analysis II, we conducted a logistic regression analysis using two models to examine the factors of patient involvement.

**Results:**

Analysis I included 277 patients. The patients' actual roles were as follows: most active (4.0%), active (10.8%), collaborative (24.6%), passive (35.0%), and most passive (25.6%). Their preferred roles were: most active (3.3%), active (18.4%), collaborative (39.4%), passive (24.5%), and most passive (14.4%). The congruence between actual and preferred roles by the kappa coefficient was 0.38. Analysis II included 218 patients. The factors for patient involvement were the clinical environment, the patient's preferred role, and the SDM-Q-9 score.

**Conclusions:**

The patients in Japan indicated a low level of decision-making involvement in physiotherapy. The patients wanted more active involvement than that required in the actual decision-making methods. The physiotherapist's practice of SDM was revealed as one of the factors related to perceptions of patient involvement in decision-making. Our results demonstrated the importance of using SDM for patient involvement in physiotherapy.

## Background

The practice of evidence-based medicine (EBM) requires the integration of the best research evidence with clinical expertise and patients' unique values and circumstances [1]. It is imperative that clinicians and patients work together to make better decisions about patient care. However, over the last few years, EBM education has focused on finding and appraising research evidence, with much less focus on how clinicians should involve their patients in treatment decision-making. As a result, we know that although physicians prefer to make shared decisions with patients, in daily clinical practice most patients are not fully involved in decisions regarding their treatment [2]. This situation is similar in the field of physiotherapy. In fact, a perception-practice gap exists in which even though roughly one-half of physiotherapists have reported favoring a shared decision model, about two-thirds typically use a rather paternalistic model in clinical practice [3, 4]. Although life-threatening decisions are rarely made in physiotherapy, respecting the autonomy and rights of the patient is an essential component of the physiotherapist’s role in supporting patients living with a disability. In light of that finding, the challenge of EBM in physiotherapy is to improve patient involvement in decision-making [5, 6].

In recent years, shared decision-making (SDM) — which integrates evidence and patients' preferences — has received much attention to address this challenge. SDM is a set of processes aimed at involving patients in health decision after the clinician and patient have discussed the available treatment options including its benefits and harms, with consideration of the patient's values, preferences, and circumstances [7]. SDM is expected to improve patients' treatment satisfaction, adherence to treatment regimens [8], and self-management skills [9]. In addition, SDM is recommended for use in situations with a high degree of uncertainty [10, 11], such as in cases of chronic disease and multimorbidity, and SDM is often indicated in physiotherapy where no established evidence for the patient's problem and multiple treatment options is available [12].

On the basis of these findings, several studies have emphasized the need for more research into the use of SDM in the field of physiotherapy, including investigations of educational methods and an organizing framework of SDM [5, 6, 13, 14]. However, the existing evidence remains insufficient to support the usefulness of SDM in physiotherapy. A fact-finding survey regarding decision-making methods has not been sufficiently investigated within the physiotherapy field. Most of the previous studies that found a lack of patient involvement in decision-making in physiotherapy were qualitative studies based on patient interviews [15], and it is not clear to what extent patients are involved or not involved in decision-making as a perception of patient involvement. In addition, the increasing number of recommendations for SDM in physiotherapy is based on research in other fields of study, and it is not yet known whether SDM as a process is related to patient perceptions of their involvement in physiotherapy. Finally, the perceived patient involvement in decision-making may be influenced by differences in culture and health care systems, such as patient age [16, 17], educational level [16–18], and the educational systems that are available regarding SDM for physiotherapists [3, 12]. We thus speculated that it is not always appropriate to apply the issues of patient involvement in decision-making observed in other countries directly to physiotherapy in Japan.

We therefore conducted the present study to: (1) clarify the issues concerning patient involvement in Japan by examining the preferences for patient involvement in decision-making and the actual decision-making methods with the use of patient perception-based quantitative assessments, and (2) examine whether SDM is related to perceptions of patient involvement in the context of physiotherapy.

## Methods

### Study design

This study was designed to be cross-sectional. The data for the study analyses were collected during the period from April 2018 to March 2019. We designed this study so that analysis I (issues related to patient involvement in decision-making) and analysis II (the relationship between SDM and patient involvement) could be performed. The reason for conducting the two analyses separately was that we anticipated a high patient burden associated with completing the questionnaire, and we thus chose this design so that the collaborating institutions could choose either analysis I only or analyses I and II. The study followed the STROBE guidelines for the reporting of observational studies.

Setting and** sample.**

**Fig. 1:**
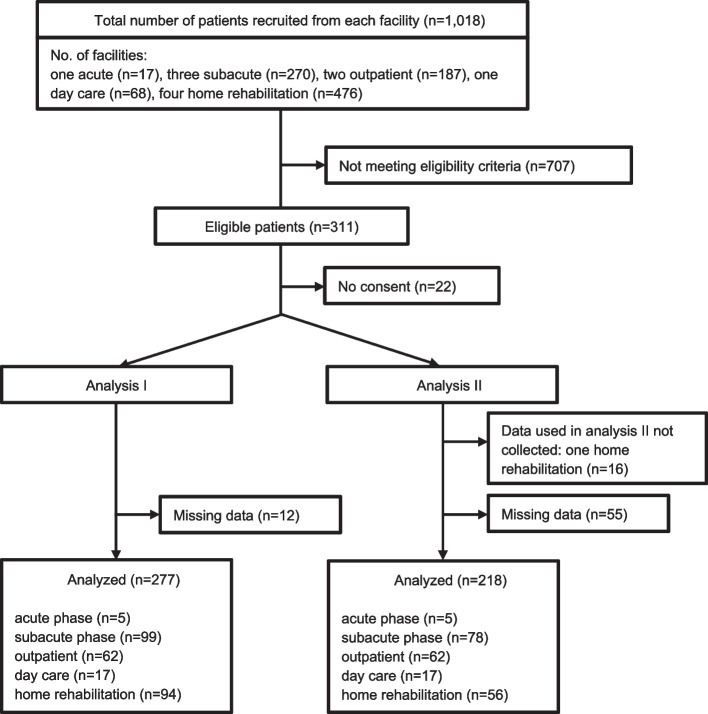
Flow chart of patient enrollment

The study participants were recruited from among acute and sub-acute inpatients and community residents receiving outpatient care, day care, and/or home rehabilitation. The eligibility criteria were patients who were (1) > 18 years of age, (2) able to speak and read Japanese, (3) undergoing physiotherapy, (4) without significant cognitive dysfunction (Hasegawa Dementia Scale-Revised [19] ≥ 21 points) and (5) with no significant hearing or visual impairment. One home rehabilitation facility was unable to collect data for use in analysis II due to the burden on patients to complete the questionnaire; the number of participants is thus slightly different between the analyses. Figure 1 shows the flow chart of patient enrollment.

The study collaborators at each facility were instructed to fully inform their eligible patients about this study and to ask informed consent for their participation. Thereafter, patients who consented to participate in the study were asked to complete all questionnaires. We recommended that study collaborators other than the physiotherapist in charge requested the patient's questionnaire responses, in order to avoid measurement bias as much as possible. However, it was difficult for the study collaborators to obtain the data of the patients who were undergoing home rehabilitation (due to human resource limitations), and the physiotherapist in charge then requested the patient's questionnaire responses. In these cases, the questionnaire was collected in a closed envelope, in order to avoid disclosure of the results to the physiotherapist in charge.

## Data collection

In earlier investigations, the factors contributing to more-passive perceptions of patient involvement in decision-making included older age [16, 17], fewer years of education [16–18], physical disabilities [20, 21], depressive symptoms [17, 22], unwillingness to be involved in decision-making [18, 23–25], and the hospital environment [26, 27]. On the basis of those studies, we used the variables of age, years of education, mobility, depression, patient's preference in decision-making, and the clinical environment in which the treatment is delivered as possible predictors of patient involvement. The clinical environments were categorized as hospital (acute and sub-acute) and community (outpatient care, day care, and home rehabilitation).

We used the Control Preference Scale (CPS) [28] to measure the type of involvement in decision making based on patient perceptions. The CPS assesses the actual decision method (the actual role) and the patient's preference (the preferred role) using a five-card system that shows how to make treatment decisions. The five cards for the patients' actual role in decision-making regarding their treatment are as follows. A: "I made the decision alone" (most active), B: "I made the decision, considering my physician's opinion" (active), C: "I shared the decision with my physician" (collaborative), D: "My physician decided, considering my preferences" (passive), E: "My physician made all the decisions" (most passive) [29]. The CPS for the patients' preferred roles are as follows. A: "I prefer to make decisions on my own" (most active), B: "I prefer to make decisions on my own after considering my physician's opinion" (active), C: "I prefer to make decisions together with my physician" (collaborative), D: "I prefer my physician to make decisions for me after considering my opinion" (passive), E: "I prefer my physician to make decisions for me" (most passive) [28]. We used the "pick one answer" approach for the patients' choice of answers on the CPS.

The CPS generally uses the following three classification methods: answers A and B as active, C as collaborative, and D and E as passive [30]. In the present study, the patients whose responses indicated an active or collaborative actual role were defined as being involved in the decision-making regarding their treatment. A Japanese version of the CPS has been developed and reported to have good reliability and validity [31].

We used the nine-item Shared Decision-Making Questionnaire (SDM-Q-9) [32] to measure the SDM process. The SDM-Q-9 is a self-report measure assessing patients' perceptions of the extent to which SDM-supportive behaviors occurred during a patient–clinician consultation. The maximum total score is 45 points, and higher scores indicate higher perceived levels of SDM during a patient-clinician consultation. In this study, as in the original version, the raw scores on the SDM-Q-9 were multiplied by 20/9 and scored out of 100. A Japanese version of the SDM-Q-9 has been developed [33] and reported to have good reliability [32] and validity [33].

Regarding decision-making among rehabilitation therapists, Weaver et al. [34] showed that SDM does not occur within a single clinical encounter; rather, it takes place as a process that unfolds across multiple clinical encounters. Therefore, the decision-making situations in the present investigation measured by the SDM-Q-9 were based on each patient's experience with the current treatment decision-making with their physiotherapist. The uses of the term "physician" in the CPS and SDM-Q-9 were modified to "physiotherapist" for this study. It is reported that assessments related to SDM can be divided into three areas: antecedents, processes, and outcomes, each with functional and operational differences. [35]. In the present study, we used the preferred role of the CPS as a patient characteristic for the desire to be involved in decision-making, the SDM-Q-9 as a set of processes aimed at involving patients in decision-making, and the actual role of the CPS as the impact of processes on patient involvement in decision making.

The physical impairment parameter that we used was mobility, which tends to cause problems in physiotherapy. The Functional Ambulation Categories (FAC) [36] walking test was used to measure the patients' mobility. We classified the patients' levels of independence into two groups: dependent in walking (FAC < 4) and independent in walking (FAC ≥ 4). The FAC is reported to have reliability and validity [37].

We used the Raskin Depression Rating Scale (RDRS) [38] to measure the patients' depression. The RDRS is a brief observer assessment in which clinicians assess the severity of three areas of depression. We classified the patients' levels of depression using 7 points as the cutoff point [39]. The RDRS was reported to be validy [40]. The FAC and RDRS, which both require observation of the patient, were measured by the patient's responsible physiotherapist in the clinical practice setting.

## Data analyses

We performed two analyses. In analysis I, we conducted a fact-finding study using the five categories (A–E) of the CPS to identify the percentages of the patients' actual and preferred roles in decision-making. To clarify whether the patients' preferences regarding their involvement in decision-making were satisfied, we also calculated the weighted kappa coefficient between the patients' actual and preferred roles. In analysis II, we conducted a logistic regression analysis using two models to examine whether the SDM-Q-9 score is related to perceptions of patient involvement as defined by the actual role of the CPS. Correlation coefficients were also calculated to examine the pattern of correlation among the variables. On the basis of previous studies, the following independent variables were included in Model 1: (1) age, (2) years of education, (3) clinical environment [hospital, community], (4) FAC [level < 4, level ≥ 4], and (5) preferred role of CPS [passive, active, or collaborative]. The RDRS was excluded from this analysis because only 6% of the patients exceeded the cutoff and no reliable depressive variables were available. We set up Model 2 by adding the SDM-Q-9 score to Model 1. It was not possible to fix the length of the episode of care (days) for each patient (e.g., initial treatment decisions). It has been suggested that good continuity of care can engender a positive patient-provider relationship, which in turn facilitate the patient's participation in SDM [41]. We therefore controlled for the number of days from the date of the current physiotherapist's charge. We also classified the patients whose responses on the CPS indicated that their actual role was passive as a non-participation group who perceived themselves as not involved in decision-making. We classified the patients whose responses on the CPS indicated that their actual role was active or collaborative as the participation group. The dependent variable was the participation group/non-participation group, as classified by the actual role in the CPS. We compared the correct classification rates and the contribution rate (Nagelkerke R^2^) between the two models. The Hosmer–Lemeshow test was used to examine the goodness of fit of the model. In this study, a list-wise method was used and all cases with missing data were omitted from the analysis. The sample size was chosen for as many patients as possible based on the possibility of performing exploratory analyses; no prior sample size estimate was performed. However, referring to earlier studies that conducted fact-finding surveys, the target number of recruits in this study was set at 300 [3, 12]. Results were considered significant at p < 0.05. The analyses were conducted using IBM SPSS Statistics, ver. 20 (IBM, Chicago, IL).

## Results

### Analysis I: Fact-finding survey of patient involvement in decision-making

Analysis I included 277 patients. The personal characteristics of the patients and physiotherapists are summarized in Tables 1 and 2, respectively. The total number of physiotherapists working in the facilities that participated in this study was 170. Of these, 91 physiotherapists were in charge of the analysis subjects, and the median number of eligible patients per physiotherapist was two.

**Table 1:** The patients' characteristics (*n* = 277)

**Characteristic**		**Analysis I**	
		**No**	**%**
Age, yrs	Mean (SD)	70.8 (13.0)	
	< 65 yrs	68	24.5
	65–74 yrs	85	30.7
	≥ 75 yrs	124	44.8
Sex	Female	166	59.9
	Male	111	40.1
Household	Alone	57	20.6
	Together	220	79.4
Education, yrs	Mean (SD)	11.9 (2.6)	
	< 10 yrs	70	25.3
	10–12 yrs	135	48.7
	≥ 13 yrs	72	26.0
Environment	Hospital	104	37.5
	Community	173	62.5
Type of disorder	Musculoskeletal disease	93	33.6
	Cerebrovascular disease	92	33.2
	Spine/spinal cord disease	37	13.4
	Neuromuscular disease	33	11.9
	Visceral disease	18	6.5
	Others	4	1.4
Length of the episode of care	Median (IQR)	105 (520)	
HDS-R	Mean (SD)	27.9 (14.6)	
FAC	Level < 4	77	27.8
	Level ≥ 4	200	72.2
RDRS	Score < 7	258	93.1
	Score ≥ 7	19	6.9

*HDS-R* Hasegawa Dementia Scale-Revised, *FAC* Functional Ambulation Categories, *RDRS* Raskin Depression Rating Scale

**Table 2:** The physiotherapists’ characteristics (*n* = 91)

**Characteristic**		**Analysis I**	
		**No**	**%**
Age, yrs	Mean (SD)	28.4 (4.8)	
Sex	Female	38	41.8
	Male	53	58.2
Working experience, yrs	Mean (SD)	5.7 (3.6)	
Highest degree	Vocational school	34	37.3
	Bachelor	50	55.0
	Graduate school	7	7.7

The results of the actual role and preferred role decision categories in the CPS are shown in Table 3. The patient numbers and percentages of the actual roles were as follows: most active, 11 (4.0%); active, 30 (10.8%); collaborative, 68 (24.6%); passive, 97 (35.0%); and most passive, 71 (25.6%). The preferred roles were as follows: most active, 9 (3.3%); active, 51 (18.4%); collaborative, 109 (39.4%); passive, 68 (24.5%); and most passive, 40 (14.4%). Among the five categories of the CPS, the actual and preferred roles matched in 138 patients (49.8%), with a weighted kappa coefficient of 0.38 (95% confidence interval [CI]: 0.30 to 0.46, p < 0.001). In addition, more patients wanted more active involvement than their perceived actual decision-making status indicated. In 101 patients (36.5%), the actual role was more passive than the preferred role; in 38 patients (13.7%), the actual role was more active than the preferred role.

**Table 3:**
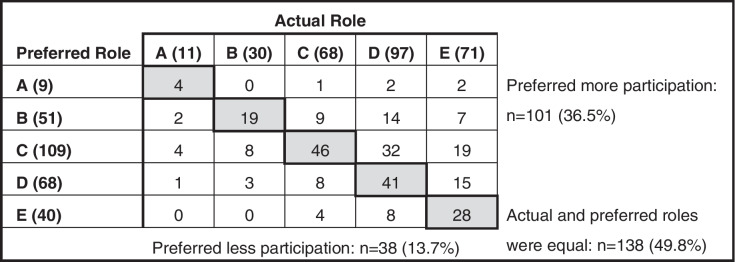
Results of the actual and preferred roles on the control preference scale

The control preference scale scores range from A (most active) to E (most passive)

The numbers in parentheses show the number of patients in each category

The bold typeface shows that the actual and preferred roles matched within the five categories of CPS (weighted kappa coefficient of 0.38, 95% CI of 0.30–0.46, *p* < 0.001)

### Analysis II: The usefulness of SDM in physiotherapy

Analysis II included 218 patients. Overall, 87 patients were classified as the participation group and 131 as the non-participation group (Table 4). The comparison of each variable between these groups revealed significant differences in the clinical environment, the patient's preferred role, and the SDM-Q-9 score. The participation group included more community-dwelling patients, showed a greater preference to be involved in decision-making, and were more likely to engage in SDM with their physiotherapist.

**Table 4:** The comparison of each variable between two groups in the analysis II

**Characteristic**		**Participation**	**Non-participation**	
		***n* = 87**	***n* = 131**	**p**^b^
Age, yrs	Mean (SD)	69.4 (13.4)	70.6 (13.2)	0.505
Education, yrs	Mean (SD)	12.0 (2.7)	11.7 (2.5)	0.484
Environment	Hospital	24	59	**0.011**
	Community	63	72	
FAC	Level < 4	23	36	1.000
	Level ≥ 4	64	95	
Preferred role	Passive	14	64	**< 0.001**
	Active/Collaborative	73	67	
SDM-Q-9 score	Median (IQR)	80 (23)	60 (32)	**< 0.001**

Bold typeface shows significant results on a level of *p* < 0.05

^a^Comparison of patients between analysis I and analysis II

^b^Comparison of participation group and non-participation group in decision making

*FAC* Functional Ambulation Categories, *SDM-Q-9* Nine-item Shared Decision-Making Questionnaire

Table 5 provides the results of the correlation matrix among the variables. Notably, the SDM-Q-9 score was weakly correlated with the patients' educational level (*ρ* = 0.22, *p* = 0.001) and clinical environment (*ρ* = 0.27, *p* < 0.001), whereas the patients' preferred role in the CPS was not correlated with any of the variables.

**Table 5:** Results of the correlation matrix among the independent variables

Variable	Education		Environment		FAC		Preferred role		SDM-Q-9	
	**coefficient**	**p**	**coefficient**	**p**	**coefficient**	**p**	**coefficient**	**p**	**coefficient**	**p**
Age	** − 0.35** ^a^	**< 0.001**	− 0.10^a^	0.133	− 0.02^a^	0.730	− 0.03^a^	0.692	− 0.08^a^	0.231
Education			**0.41**^a^	**< 0.001**	0.12 ^a^	0.071	0.04^a^	0.456	**0.22**^a^	**0.001**
Environment ^c^					**0.27** ^b^	**< 0.001**	0.05 ^b^	0.506	**0.27**^a^	**< 0.001**
FAC^d^							− 0.01^b^	0.973	0.10^a^	0.158
Preferred role^e^									0.05 ^a^	0.453

Bold typeface shows significant results on a level of *p* < 0.05

^a^Spearman’s rank correlation coefficient

^b^Phi coefficient

^c^Environment; 0 = hospital, 1 = community

^d^FAC; 0 = level < 4, 1 = level ≥ 4

^e^Preferred role; 0 = passive, 1 = active/collaborative

*FAC* Functional Ambulation Categories, *SDM-Q-9* Nine-item Shared Decision-Making Questionnaire

Table 6 provides the results of the logistic regression analysis. In Model 1, the clinical environment (odds ratio [OR] 3.25, 95%CI: 1.50–7.05, *p* = 0.003) and the patient's preferred role (OR 5.21, 95%CI: 2.63–10.32, *p* < 0.001) were revealed as significant predictors (percentage of correct classifications, 69.3%; Nagelkerke R^2^ = 0.206). In Model 2, the clinical environment (OR 2.66, 95%CI: 1.15–6.19, *p* = 0.023), the patient's preferred role (OR 6.28, 95%CI: 3.00–13.18, *p* < 0.001), and the SDM-Q-9 score (OR 1.05, 95%CI: 1.03–1.06, *p* < 0.001) were revealed as significant predictors (percentage of correct classifications, 73.4%; Nagelkerke R^2^ = 0.354). These results indicated that the perceptions of patient involvement in decision-making concerning the physiotherapy was attributable to (1) their clinical environment being in the community, (2) their own willingness to be involved, and (3) the physiotherapist's implementation of SDM.

**Table 6:** Results of the two logistic regression models for predicting patient involvement

**Summary of independent variables for patient involvement in decision-making (Model 1)**					
**Variable**	**Coefficient (B)**	**SE**	**Wald χ2**	**OR (95%CI)**	**p**
Age, yrs	− 0.009	0.012	0.588	0.99 (0.97–1.02)	0.443
Education, yrs	− 0.055	0.068	0.647	0.95 (0.83–1.08)	0.421
Environment (hospital)				1	
(community)	**1.179**	**0.395**	**8.900**	**3.25 (1.50–7.05)**	**0.003**
FAC (level < 4)				1	
(level ≥ 4)	− 0.108	0.355	0.092	0.90 (0.45–1.80)	0.762
Preferred role (passive)				1	
(active/collaborative)	**1.652**	**0.348**	**22.47**	**5.21 (2.63**–**10.32)**	**< 0.001**
Correct classification rate	69.3%				
Nagelkerke R^2^	0.206				
Hosmer–Lemeshow test	*p* = 0.186				
**Summary of independent variables for patient involvement in decision-making (Model 2)**					
**Variable**	**Coefficient (B)**	**SE**	**Wald χ2**	**OR (95%CI)**	**p**
Age, yrs	− 0.014	0.013	1.189	0.99 (0.96–1.01)	0.276
Education, yrs	− 0.110	0.076	2.103	0.90 (0.77–1.04)	0.147
Environment (hospital)				1	
(community)	**0.979**	**0.431**	**5.174**	**2.66 (1.15–6.19)**	**0.023**
FAC (level < 4)				1	
(level ≥ 4)	− 0.192	0.387	0.245	0.83 (0.39–1.76)	0.621
Preferred role (passive)				1	
(active/collaborative)	**1.838**	**0.378**	**23.65**	**6.28 (3.00–13.18)**	**< 0.001**
SDM-Q-9 (score)	**0.044**	**0.009**	**23.90**	**1.05 (1.03–1.06)**	**< 0.001**
Correct classification rate	73.4%				
Nagelkerke R^2^	0.354				
Hosmer–Lemeshow test	*p* = 0.425				

Bold typeface shows significant results at *p* < 0.05

*FAC* Functional Ambulation Categories, *SDM-Q-9*: Nine-item Shared Decision-Making Questionnaire

## Discussion

We sought to clarify the issues of patients' involvement in decision-making in the context of Japanese physiotherapy and to determine whether SDM is related to perceptions of patient involvement. The overall results demonstrated that (1) patients in Japan are less involved in decision-making in physiotherapy than they would like to be, and (2) SDM is one of the factors associated with perceptions of patient involvement. The analysis I results shown in Table 3 revealed that the actual roles perceived by patients were as follows: the passive role was the most common response by the patients at ~ 60%, compared to ~ 25% for a collaborative role and ~ 15% for an active role. From this result, we clarified that patients in Japan are not closely involved in the decision-making in their physiotherapy. Regarding the patients' perceptions of participation in physiotherapy, Schoeb et al. [15] reported varying patients' preferences in decision-making, but infrequent participation in actual decision-making, similar to the present study. However, most of the studies that investigated patients' perceptions of their involvement in decision-making used qualitative analyses [13, 15], and the extent of the patients' involvement have not been shown until now. A key strength of our study is that it reveals the reality of patient involvement in decision-making in physiotherapy by using a patient perception-based quantitative assessment. Our findings are important in publicizing the issue of inadequate involvement in decision-making in physiotherapy from the patient's perspective.

We observed that the congruence between the patients' actual and preferred roles in this study was 49.8%, but the weighted κ coefficient was low, and 36.5% of the patients wanted more active involvement. In this regard, Schoeb et al. [15] reported that patients do play a passive role in decision-making in the real world, even though they want an active role in decision-making. Our present findings partially support this discrepancy in patient preference, which was obtained in a qualitative study. That is, some patients showed a discrepancy between their actual and preferred roles, but roughly half of the patients had matching actual and preferred roles. In addition, many of the matched patients had a passive preferred role, and some patients did not desire an active role. Several studies have reported that not all patients want an active role in decisions regarding the content of the rehabilitation sessions, and some want a passive role [42, 43]. The results of this study demonstrate, in simple results, the complex realities of patient preferences reported in qualitative studies.

It is important to interpret our present findings in conjunction with those of the existing qualitative studies in clinical practice. Although we indicated earlier the issue of insufficient involvement in decision-making, we need to understand that there is not a general problem with decision-making involvement within the field of physiotherapy; rather, patients who wish to be involved in decision-making are experiencing discrepancies. In addition, the results of qualitative studies indicate that among patients who want to be more involved in decision-making, there is a need for more interaction between the patients and their physiotherapists, including explanations by the physiotherapists that affirm their own role and involvement in decision-making, the building of partnerships, and listening to the patients' opinions about treatment plans [15]. Integrating both of these findings would enable a deeper understanding of patient involvement in the physiotherapy field. Our results clarify the challenges of patient involvement in physiotherapy in Japan and indicate that these challenges are similar across cultures and health care systems.

In analysis II, the clinical environment, the patients' preferred role, and SDM were selected as factors related to perceptions of patient involvement in treatment decision-making. The inpatient environment in rehabilitative medicine requires compliance with facility rules and physician orders, which can be a barrier to involvement in decision-making [26, 27]. Qualitative studies focusing on physiotherapists [24, 25] and patients [44, 45] have also shown that factors at the early stages of illness require less patient involvement in decision-making due to the patients' lack of treatment knowledge and experience. We thus speculate that this may help explain why the inpatients in the present study had less involvement in decision-making compared to the community-dwelling patients. Passive patients who do not like to be involved in decision-making were reported as one of the barriers to involvement in physiotherapy [24, 25], and our present findings complement those studies. As a factor related to preference in decision -making, Bernhardsson et al. [43] observed that some patients did not have a clear treatment preference and expressed great trust that their physiotherapist would be competent to know the best options and make the best decisions, thus preferring to leave the decisions to the physiotherapist. Holliday et al. [44] identified the following factors for patient participation in goal setting: clarifying the patient's role in the rehabilitation process, discussions about the mechanisms of impairment and recovery, determining the patient's prior experience of goal setting, and clarifying expectations for participation in goal setting. An implication of these findings is that in addition to conducting an assessment of a patient's preferences in treatment decision-making, it is important to check in advance whether the patient is ready to be involved in decision-making; for example, whether the patient has undue expectations of the physiotherapist or whether the patient has the necessary knowledge to be involved in decision-making. In trying to encourage patient involvement in decision-making, we may need to recognize the importance of this discussion and patient preparation and allocate some time for this work.

We also observed that (i) Model 2 showed a significant association with SDM in addition to the clinical environment and the patient's preferred role, and (ii) Model 2 had a higher contribution than Model 1. Pollard et al. [18] reported three types of factors involved in decision-making: physician-, patient-, and condition-related factors. We observed significant associations between SDM as a therapist-related factor, the patient's preference as a patient-related factor, and the clinical environment as a condition-related factor. Thus, when clinicians seek to support patient involvement in decision-making, all of this information must be integrated (not just SDM). Interestingly, the odds ratio for the preferred role of CPS was higher in Model 2 than in Model 1. The results in Table 5 show that the SDM-Q-9 score was correlated with several variables, whereas the preferred role in the CPS was not correlated with any of the variables. We thus consider that the preferred role in the CPS was more independent throughout the model.

Some of the variables that we examined did not show significant associations with the patients' involvement in decision-making. Regarding patients' educational level, Brom et al. [17] reported that lower educational levels were associated with a more passive role and thus considered a barrier to patients' involvement in decision-making. Japan has a compulsory education system, and the present patients had a higher level of education compared to a previous study [16] (≤ 9 years of education: 25.3% vs 65.2%). The impact of the education level on patient involvement may thus be insignificant in Japan. Regarding physical disability, Fried et al. [21] reported that having multiple conditions one of the factors that make it difficult for older patients to be involved in decision-making. We thus speculated that the present patients with a dependent gait would be less likely to be involved in the decision-making about their treatment. However, our analyses revealed no association between patient involvement and gait independence. The present patients were without cognitive dysfunction, and they may have been able to make decisions on their own even if their physical disabilities were severe. In addition, compared to primary care, physiotherapy poses less risk to the patient in decision-making, so patients may be more likely to be involved. Regarding age, it was reported that older people have a more passive role in decision-making [16, 17]. Our present patient population was biased toward the elderly (mean age ~ 71 years), and a valid analysis-based conclusion could thus not be reached.

Our study has some limitations. First, regarding the patient recruitment, we used convenience sampling from multiple centers, but acute-phase care and day care settings were limited to recruitment from a single facility. In addition, many data were missing in analysis II. This may have been influenced by patient concerns about scoring the SDM of the assigned physiotherapist. Possible selection bias should thus be considered. Second, there are challenges related to the measurement of SDM processes. In this study, the measurement of SDM was based on each patient's experience with current treatment decision-making with their physiotherapist. However, the patients' most recent decision-making situation was not clearly defined, which may have resulted the recall bias. Third, we used a self-reported SDM assessment, and it should thus be noted that the implementation of SDM is not an objective behavior of physiotherapists, but rather a perception based on patient experience. In this regard, it is known that there is no correlation between the SDM-Q-9, which measures a patient's perception, and the OPTION scale, which measures the physician's behavior through a third party [46]. It is thus not clear from our present findings whether objective SDM practices by physiotherapists are related to the patients' involvement.

## Conclusion

This study is the first to report the extent of patient involvement in treatment decision-making based on their perceptions within the field of physiotherapy. Our results demonstrated that patients in Japan are less involved in physiotherapy decision-making than they would like to be, revealing the same challenges as in other countries across cultures and healthcare systems. We also obtained evidence that the physiotherapist's practice of SDM is an important factor related to perceptions of patient involvement in decision-making. We believe that the practice of SDM by physiotherapist is a useful method to reduce the perception of patient involvement in unmet decision making in the physiotherapy field.

## Data Availability

All materials and data of this study are available from the corresponding author on request.

## Abbreviations

SDM: Shared decision making
CPS: Control preference scale
SDM-Q-9: Nine-item shared decision-making questionnaire
EBM: Evidence-based medicine
FAC: Functional ambulation categories
RDRS: Raskin depression rating scale
